# Sesamin Promotes Neurite Outgrowth under Insufficient Nerve Growth Factor Condition in PC12 Cells through ERK1/2 Pathway and SIRT1 Modulation

**DOI:** 10.1155/2020/9145458

**Published:** 2020-03-26

**Authors:** Sasimol Udomruk, Chayanut Kaewmool, Thanyaluck Phitak, Peraphan Pothacharoen, Prachya Kongtawelert

**Affiliations:** Thailand Excellence Center for Tissue Engineering and Stem Cells, Department of Biochemistry, Faculty of Medicine, Chiang Mai University, Chiang Mai 50200, Thailand

## Abstract

The promotion of neurogenesis can be a promising strategy to improve and restore neuronal function in neurodegenerative diseases. Nerve growth factor (NGF) plays a key role in neurite outgrowth and synaptic formation during brain repair stage. Nowadays, there are several studies on the developing methods to enhance the endogenous NGF activity for treatment and restore the neuronal function. In this study, the potentiating effect of sesamin, a major lignan in sesame seeds (*Sesamum indicum*) and oil, on NGF-induced neurogenesis and its involved mechanisms were firstly reported. Sesamin effectively enhanced the PC12 neuron-like cell differentiation and neurite length under insufficient conditions of NGF. The neuronal markers including synaptophysin and growth-associated protein-43 along with the synaptic connections were significantly increased in combination treatment between sesamin and NGF. Moreover, sesamin also increased the level of phospho-ERK1/2 and SIRT1 protein, an important regulatory protein of the neurogenesis process. The neurogenesis was blocked by the specific SIRT1 inhibitor, JGB1741, suggesting that the neuritogenic effect of sesamin was associated with SIRT1 protein modulation. Taken together, the potentiating effect of sesamin on NGF-induced neurogenesis in this finding could be used for alternative treatment in neurodegenerative diseases, including Alzheimer's disease.

## 1. Introduction

Neuronal loss and synaptic dysfunction are the main pathological features which linked to cognitive impairments in neurodegenerative diseases such as Alzheimer's disease (AD) and Parkinson's disease (PD) [1]. Developing therapeutic approaches that target on the promotion of neurogenesis is a greatly promising strategy for the neuronal regeneration and reconstruction of synaptic networks in neurodegenerative diseases.

Nerve growth factor (NGF), a member of the neurotrophic factor family, is a key modulator in neuronal differentiation, promoting neurite outgrowth and synaptic connection. The binding of NGF and tropomyosin-receptor-kinase A (TrkA) receptor stimulates downstream signaling pathways, resulting in expression of genes essential for neuronal differentiation such as growth-associated protein-43 (GAP43) and synaptophysin (SYP) via the extracellular signal-regulated kinase (ERK1/2-MAPK) signaling pathway [2]. However, the mature NGF level is reduced in neurodegenerative diseases, especially AD, leading to NGF insufficiency in patients [3]. Currently, NGF supplementation becomes to be an interesting option to restore neuronal function. Nonetheless, the delivery of NGF into the brain is limited by the blood-brain barrier (BBB) [4]. However, the long-term NGF administration by ICV injection was associated with the remarkable side effects [5]. Thus, the investigation of molecules that can enhance the endogenous NGF function has been proposed to be a promising alternative strategy to overcome the insufficient conditions of NGF. Nowadays, several natural products have been reported to potentiate the action of NGF to induce neurogenesis [6].

Sesamin, a major lignan compound found in sesame seeds and oil, is considered to possess several bioactivities to be beneficial for human health and is widely used as a supplement, especially in Asian countries. The neuroprotective effect of sesamin was reported in various studies [7–9]. Our previous study also reported that sesamin inhibited microglial activation and protected neuronal cell death induced by neurotoxic factors [9]. However, its effect on neurogenesis is not well established. In this study, the neuritogenic effect of sesamin was firstly demonstrated by using the PC12 differentiation model. The pheochromocytoma cell line (PC12) can be differentiated into neuron-like cells by NGF stimulation and shared the morphological function similar to neurons. Thus, PC12 cells are usually used as a model system for neurogenesis or neuronal differentiation [10]. Here, we demonstrated that sesamin potentiated NGF activity to increase the percentage of neuron-like cell differentiation, neurite length, and synaptic connections in PC12 cells, *in vitro* model. These effects were involved in the enhancement of the ERK1/2-MAPK signaling pathway and modulation of SIRT1 protein. Taken together, this finding suggests that sesamin might be a potential therapeutic compound for neuronal functions repair in neurodegenerative diseases by promoting neurogenesis.

## 2. Materials and Methods

### 2.1. Sesamin Preparation

Sesame seeds were amassed from Lampang, Thailand. The voucher specimens (BKF no. 138181) were approved by the National Park, Wildlife and Plant Conservation Department, Ministry of Natural Resources and Environment, Bangkok, Thailand. Sesamin was prepared as previously described according to Phitak's and Kansai's reports [11, 12].

### 2.2. Cell Culture

Rat pheochromocytoma (PC12) cells were obtained from CLS Cell Lines Service GmbH (Germany). Cells were grown in DMEM (Gibco, NY, USA) supplemented with 10% HS and 5% FBS (Gibco, NY, USA). They were maintained in 100 units/ml penicillin and 100 *μ*g/ml streptomycin, in a tissue culture incubator at 37°C with 5% CO_2_. The growth medium was changed three times a week.

### 2.3. Cell Viability Assay

Cell viability was measured by using MTT (3-[4, 5-dimethylthiazol-2-yl]-2, 5-diphenyl tetrazolium bromide) assay. Briefly, cells were incubated with 100 *μ*l of 0.5 mg/mL MTT solution (Sigma-Aldrich, St. Louis, MO, USA) in a final concentration, at 37°C for 4 h. The culture medium was removed, and formazan crystals were solubilized in 100 *μ*l dimethyl sulfoxide (DMSO). The reduction of MTT was measured at 540 nm using a microplate reader spectrophotometer. The results were expressed as the percentages of cell viability by the following equation: percentage of cell viability = (OD of sample/OD of control) × 100.

### 2.4. Quantification of Neurite Outgrowth

To evaluate the effects of NGF and/or sesamin on neurite outgrowth, PC12 cells were seeded at 1.5 × 10^5^ cells/well in 6-well plates coated with 0.1 mg/mL poly-L-lysine (Sigma-Aldrich, MO, USA). PC12 differentiation was stimulated by NGF (R&D system, MN, USA) under 1% HS and 0.5% FBS in DMEM. Neurite outgrowth was observed in eight randomly selected fields using an inverted phase-contrast microscope. The cell that exhibits the least one neurite with a length that longer than the diameter cell body was identified as the differentiated cell. The number of differentiated cells was expressed as a percentage of the total cell number. Moreover, the longest neurite length of the differentiated cell was scaled by AxioVision software. To investigate the effect of sesamin on neurite outgrowth, the JGB1741, a specific SIRT1 inhibitor from APExBIO Technology LLC (Houston, TX, USA) was used.

### 2.5. Gene Expression

To examine the gene expression level, total cellular RNA was isolated using the illustra RNAspin Mini kit (GE Healthcare Europe GmbH, Freiburg, Germany). 500 ng of total RNA was converted to cDNA by iScript™ (Bio-Rad, Hercules, CA, USA) following the manufacturer's instructions. The PCR was performed with 45 cycles of 5 s at 95°C, 10 s at 60°C, and 30 s at 72°C by Applied Biosystems 7500/7500 Fast Real-Time PCR System using SensiFAST™ SYBR® Lo-ROX (Bio-Rad, Hercules, CA, USA). Synaptophysin (*SYP*), growth-associated protein-43 (*GAP43*), and sirtuin-1 (*SIRT1*) genes were measured to determine the neuronal differentiation. The relative expression level of each gene was normalized with the beta-actin (*ACTB*) gene by using the 2^(−ΔΔC(T))^ method [13]. The primers used in the experiment are shown in Table 1.

### 2.6. Western Blot Analysis

The 30 *μ*g proteins of cell lysate were separated on 10% SDS-PAGE and transferred to nitrocellulose membrane (GE Healthcare, Germany). After blocking, membranes were probed with primary antibodies against a 1 : 500 dilution of synaptophysin (ab178412) (Abcam, Cambridge, UK), or 1 : 1000 dilution of GAP43 (8945s), p-Akt (9271s), p-ERK (9101s), total Akt (9272s), total ERK (9102s), SIRT1 (9475s), and *β*-actin (4970s) (Cell Signaling, MA, USA) at 4°C, overnight. The membranes were washed and incubated with a horseradish peroxidase-conjugated secondary antibody (7074S) (Cell Signaling, MA, USA) in the 1 : 1000 dilution for 1 h at room temperature. The chemiluminescence signal was visualized using the SuperSignal West Femto Maximum Sensitivity Substrate kit (Thermo Fisher Scientific, USA). The band intensity was quantified by TotalLab TL120 software.

### 2.7. Immunofluorescence Staining

The PC12 cells were seeded at 1.5 × 10^5^ cells/well on poly-L-lysine-coated 10 mm ∅ glass slides (Menzel™ Microscope Coverslips). The cells were treated with NGF alone and cotreated with sesamin. Then, cells were fixed with 4% paraformaldehyde at room temperature for 15 min and permeabilized with 0.1% Triton X-100-PBS for 10 min. After blocking with 1% BSA, 22.52 mg/mL glycine in PBS-T for 1 h, cells were incubated with anti-SYP antibody (1 : 500) or anti-SIRT1 antibody (1 : 400) at 4˚C, overnight. Then, goat anti-rabbit IgG (Alexa Fluor® 488) (ab150077) (Abcam, Cambridge, UK) was probed (1 : 1000) and incubated for 1 h and counterstained with DAPI. Cells were visualized by a fluorescence microscope (Zeiss Axio Scope A1, Germany). The quantitative analysis of fluorescence intensity was measured by ImageJ software.

### 2.8. Statistical Analysis

All experiments were repeated three replications. The data were represented as the mean ± SD. Statistical analyses were performed using SPSS 12.0 software. The significant differences were determined using one-way ANOVA with Tukey HSD's post hoc test. A value of *p* < 0.01 and *p* < 0.05 were considered statistically significant.

## 3. Results

### 3.1. Effect of Sesamin on the Cell Viability of PC12 Cells

**Figure 1:**
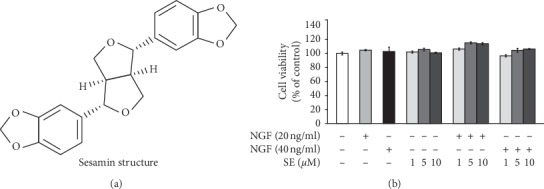
Effect of sesamin and nerve growth factor (NGF) on PC12 cell viability. (a) Structure of sesamin. (b) To study the effect of sesamin or NGF on cell viability, PC12 cells were seeded at 1 × 10^4^ cells/well in 96-well plate. Then, cells were treated with sesamin alone at 1, 5, and 10 *μ*M or with NGF at 20 and 40 ng/ml. To investigate the cytotoxic effect of combination treatment between sesamin and NGF, cells were cotreated with sesamin (1, 5, and 10 *μ*M) and NGF (20 or 40 ng/ml). After 24 h incubation, PC12cell viability was measured by MTT assay. Data represent the means ± SD of three independent experiments.

The effect of the natural compound on cell viability is crucial to ascertaining the nontoxic concentration. The results showed that sesamin alone, NGF alone, and the combination treatment had no apparent cytotoxic effect on PC12 cells (Figure 1(a) and 1(b)). Thus, these conditions were utilized in subsequent experiments.

### 3.2. Effect of Sesamin on NGF-Induced Neurite Outgrowth in PC12 Cells

**Figure 2:**
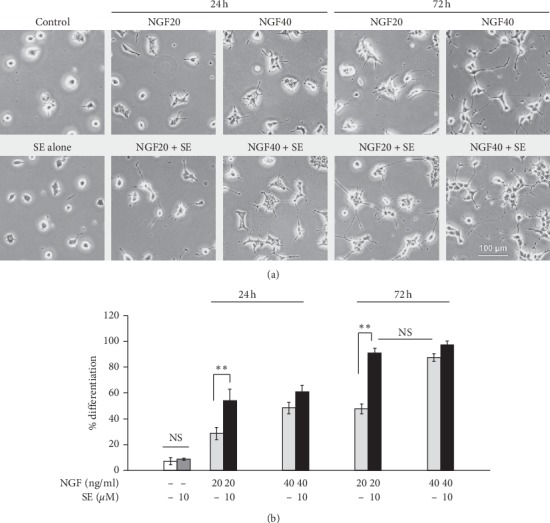
Effects of sesamin on the induction of morphological differentiation by NGF stimulation. The PC12 cell differentiation was stimulated with NGF at 20 ng/ml (low concentration) or 40 ng/ml (high concentration) under cotreatment with/or without 10 *μ*M sesamin. (a) The morphological change was observed under an inverted phase-contrast microscope at 24 and 72 h after treatment. (b) The bar graph showed the calculation of percent differentiation. Data represent the means ± SD of three independent experiments. ^*∗∗*^*p* < 0.01 compared with NGF alone. SE: sesamin; NGF: nerve growth factor.

To screen the potential effect of sesamin on NGF-induced neurite outgrowth, PC12 cells were treated with NGF alone or plus sesamin at different concentrations of NGF (20 or 40 ng/ml) and incubation times (24 or 72 h). The results showed that the percentage of PC12 differentiation in the 20 ng/ml NGF-treated group was lower than that in the 40 ng/ml NGF-treated group in both 24 and 72 h incubation times. These results represented the insufficient effect of NGF in a low dose (20 ng/ml). Interestingly, the differentiation of insufficient NGF condition was markedly increased when combined with sesamin treatment in both 24 and 72 h. The potentiation level of combination treatment can be equivalent to an effective dose of NGF at 40 ng/ml (Figures 2(a), 2(b)). However, the effect of sesamin combined with a high dose of NGF slightly affected differentiation. As the differentiation was completely induced at a high dose of NGF, the effect of sesamin could not be observed. In this study, NGF at 20 ng/ml (insufficient NGF condition) and 72 h incubation time were chosen to investigate the effect of sesamin potentiated NGF-induced neurite outgrowth.

**Figure 3:**
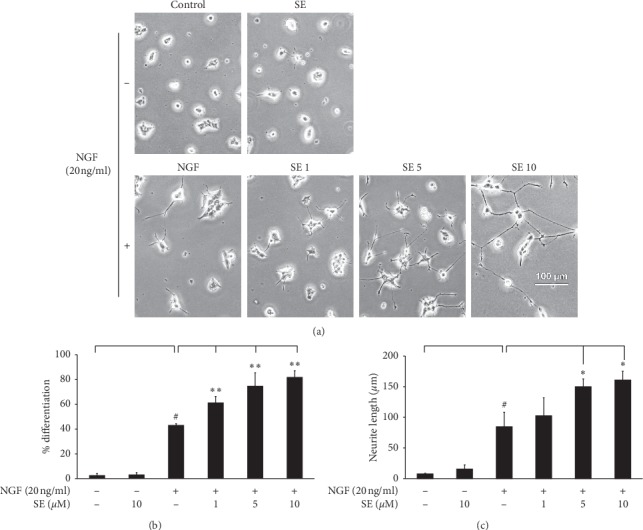
Dose-dependent effect of sesamin on morphological differentiation and neurite length by NGF stimulation. The PC12 cells enhanced the differentiation with NGF at 20 ng/ml and cotreated with/or without sesamin at various concentrations (1, 5, and 10 *μ*M) for 72 h. (a) The morphological change was observed under an inverted phase-contrast microscope. (b) The bar graph showed the calculation of differentiation percentage. (c) Neurite length was quantified by AxioVision Rel.4.8. Data represent the means ± SD of three independent experiments. ^#^*p* < 0.05 compared with the control group; ^*∗*^*p* < 0.05 and ^*∗∗*^*p* < 0.01 compared with the NGF alone treatment group. SE: sesamin; NGF: nerve growth factor.

The dose-dependent effect of sesamin on differentiation was further examined. The combination treatment exhibited proportion of cells with neurites increased to 61.5%, 74%, and 82%, respectively (Figure 3(b)) when compared with NGF alone (43%). Moreover, the elongation of neurite outgrowth can be seen in the combination treatment in a dose-dependent manner of sesamin (Figure 3(c)). However, we found that sesamin alone did not affect neurogenesis without NGF. These results strongly indicate that the neuronal differentiation induced by a low dose of NGF can be potentiated by sesamin.

### 3.3. Effect of Sesamin on the Expression of Neuronal Markers in PC12 Cells

**Figure 4:**
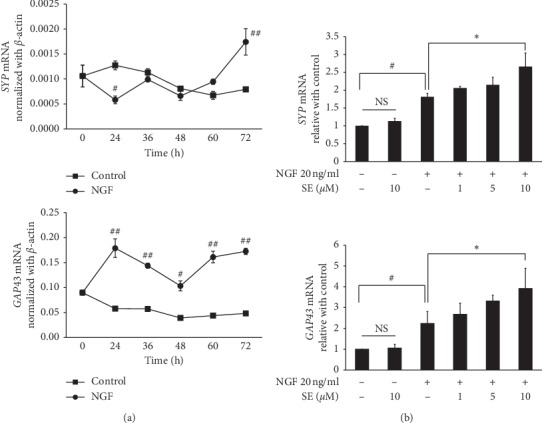
Effect of NGF and sesamin on neurite outgrowth marker gene expression. (a) To investigate the gene expression time period of NGF induction, PC12 cells were stimulated with NGF at 20 ng/ml for 0, 24, 36, 48, 60, and 72 h. (b) To demonstrate the effect of sesamin, cells were cotreated with or without 1, 5, and 10 *μ*M of sesamin and 20 ng/ml of NGF for 72 h. The mRNA level of neurite outgrowth markers (*SYP* and *GAP43*) was determined by real-time PCR. Relative mRNA expression was normalized using *ACTB* expression. Data represent the means ± SD of three independent experiments. ^#^*p* < 0.05 compared with the control group; ^*∗*^*p* < 0.05 and ^*∗∗*^*p* < 0.01 compared with the NGF alone treatment group. SE: sesamin; NGF: nerve growth factor.

**Figure 5:**
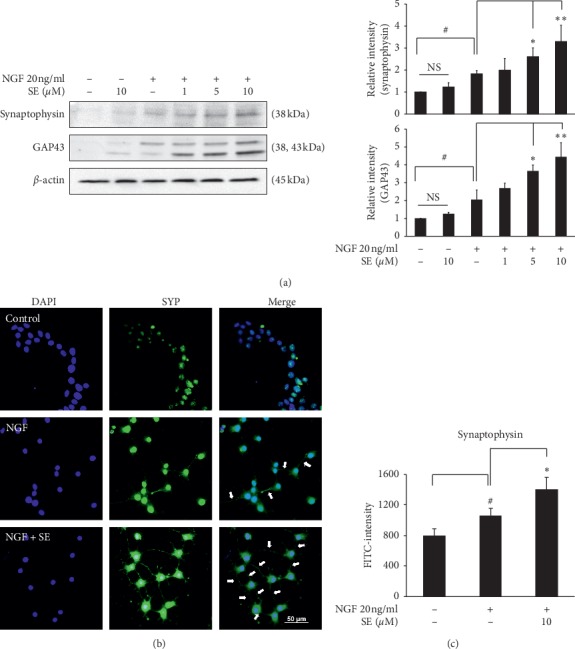
Effect of sesamin enhanced NGF function on neurite outgrowth markers and synaptic connection. After cotreated with or without 1, 5, and 10 *μ*M of sesamin and 20 ng/ml of NGF for 72 h, (a) cell lysates were collected to investigate the synaptophysin and GAP43 protein levels by western blot analysis. The *β*-actin was used as an internal control and band intensities were quantified by using TotalLab TL120 software. (b) The distribution of synaptophysin protein and the synaptic connection were investigated by immunostaining. (c) The bar graph showed the fluorescent intensity of synaptophysin. Data represent the means ± SD of three independent experiments. ^#^*p* < 0.05 compared with the control group; ^*∗*^*p* < 0.05 and ^*∗∗*^*p* < 0.01 compared with the NGF alone treatment group. SE: sesamin; NGF: nerve growth factor.

GAP43, a protein expressed at neuronal growth cone, plays key roles on the regulation of axonal elongation, while synaptophysin is an essential synaptic vesicle protein. These proteins are widely used as the neurogenesis and synaptic formation markers. The response of *SYP* and *GAP43* gene expression on NGF treatment alone was firstly investigated to demonstrate the gene expression pattern. After NGF stimulation, the *SYP* was increased in a time-dependent manner and the highest level was found at 72 h. Meanwhile, the upregulation of *GAP43* was initially observed at 24 h and maintained the level until 72 h (Figure 4(a)). Thus, 72 h incubation was used as the optimal time to investigate both genes. Next, we observed the effect of sesamin on neurite outgrowth markers. Interestingly, the combination treatment with 10 *μ*M sesamin significantly increased the *SYP* and *GAP3* mRNA levels (Figure 4(b)). Likewise, the western blot analysis revealed that GAP43 and SYP protein levels were significantly increased by the combination treatment after 72 h (Figure 5(a)). These results suggest that sesamin potentiates NGF-induced neuronal marker expression.

### 3.4. Effect of Sesamin on Synaptic Connections

Synaptic connectivity is necessary for the transmission of information. The distribution of synaptophysin at growing neurites and growth cones is important for synaptic connections. The number of synaptic connections can be quantified by observing the distribution patterns of synaptophysin [14]. Fluorescence immunostaining showed that the combination treatment exhibited the distribution of synaptophysin on neurites. Cells in this group obviously presented a large number of synaptic connections, which were shown as the linkage between neurites of each cell (the connection was pointed out by white arrow) (Figure 5(b)). Conversely, cells treated with NGF alone showed a lower number of connections. Moreover, the synaptophysin-fluorescent intensity was significantly increased in combination treatment when compared with NGF alone (Figure 5(c)), suggesting that sesamin not only promotes neurite outgrowth but also enhances the formation of synaptic connections under insufficient NGF condition.

### 3.5. Effect of Sesamin on the Signaling Pathway

**Figure 6:**
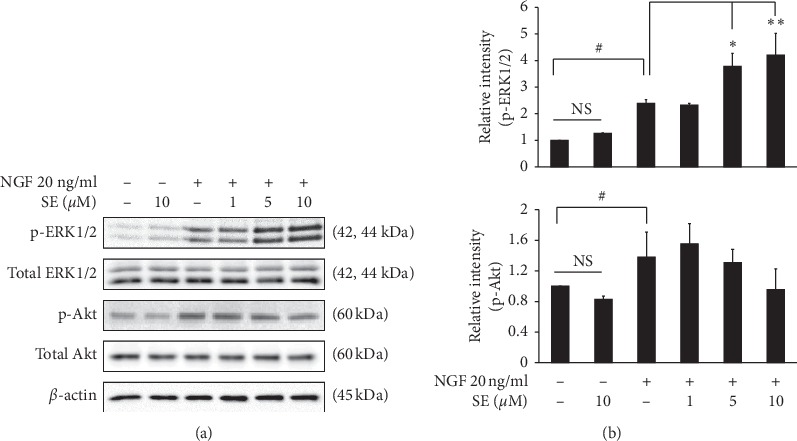
Effects of combination treatment on signaling pathways. To investigate the effect of sesamin on phosphorylation of ERK1/2 and Akt pathway-dependent NGF stimulation, PC12  cells were cotreated with various concentrations of sesamin (1, 5, and 10 *μ*M) and NGF 20 ng/ml for 4 h. (a) The levels of p-ERK1/2 and p-Akt were measured by western blot analysis and normalized by the total form of ERK1/2 and Akt, respectively. (b) The band intensity was quantified by TotalLab TL120. Data represent the means ± SD of three independent experiments. ^#^*p* < 0.05 compared with the control group; ^*∗*^*p* < 0.05 and ^*∗∗*^*p* < 0.01 compared with the NGF alone-treated group. SE: sesamin; NGF: nerve growth factor.

This study investigated whether ERK1/2 and Akt pathways were related to the neurite outgrowth induced by the combination treatment. The result showed that the combination treatment was significantly potentiated on ERK1/2 activation in a dose-dependent manner compared with NGF alone, while sesamin treatments showed any effect on the phosphorylated Akt (Figures 6(a) and 6(b)). These results demonstrate that the effect of the combination treatment on neurite outgrowth is associated with the ERK1/2 activation.

### 3.6. Effect of Sesamin on SIRT1

**Figure 7:**
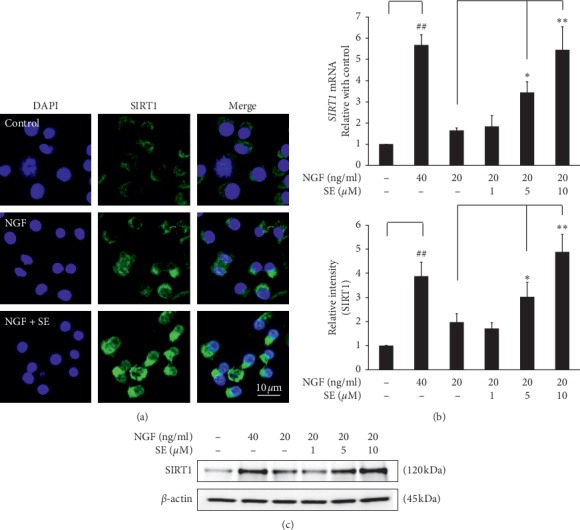
Effects of sesamin on SIRT1 expression and intracellular localization. (a) PC12 cells were treated with 20 ng/ml of NGF alone or combined with sesamin 10 *μ*M for 72 h and then the SIRT1 localization was observed by immunofluorescence staining. Next, PC12 cells were treated with 20 ng/ml of NGF alone, 40 ng/ml of NGF alone, and combination treatment with various sesamin concentrations (1, 5, and 10 *μ*M). (b) After 24 h, the mRNA level of SIRT1 was determined by real-time PCR. (c) At 72 h, the SIRT1 protein was measured by western blot analysis and the band intensity was quantified by TotalLab TL120. Data represent the means ± SD of three independent experiments. ^##^*p* < 0.05 compared with the control group; ^*∗*^*p* < 0.05 and ^*∗∗*^*p* < 0.01 compared with the NGF alone-treated group. SE: sesamin; NGF: nerve growth factor.

Sirtuin-1 (SIRT1), also known as NAD+-dependent deacetylase, is one of the important regulatory proteins in the neurogenesis process [15, 16]. Interestingly, the upregulation of SIRT1 was implicated in potentiating NGF-induced neurogenesis in PC12 cells [17]. Thus, we further investigated whether the synergistic effect of sesamin on NGF-induced neurite outgrowth is associated with SIRT1 protein modulation. The result showed that the expression of SIRT1, in both mRNA and protein levels, was markedly increased when exposed to high NGF (40 ng/ml), while it was only slightly increased in low NGF condition. Furthermore, the NGF-mediated upregulation of both gene and protein levels of SIRT1 was augmented when combined with sesamin, in dose-dependent manner (1, 5, and 10 *μ*M) (Figures 7(b) and 7(c)). Moreover, the intracellular localization of SIRT1 was also observed by immunostaining. The result showed that SIRT1 protein was upregulated in both cytoplasm and nucleus of the PC12 cells exposed to combination treatment, compared with low NGF alone (Figure 7(a)). Interestingly, the increasing level of SIRT1 by combination treatment was correlated with percent differentiation and neurite length.

**Figure 8:**
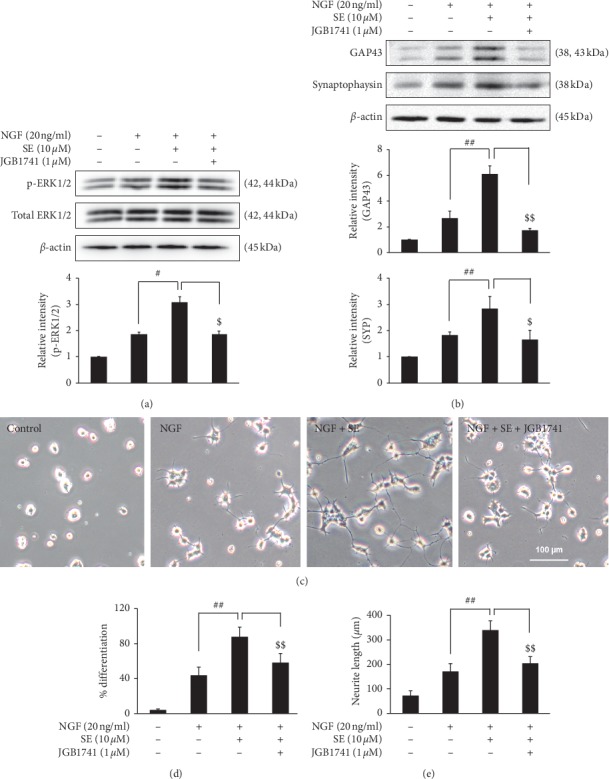
To confirm the correlation of SIRT1 effect and neuronal differentiation, cells were treated with JGB1741, the selective SIRT1 inhibitor under combination treatment. (a) Cell lysates were collected at 4 h to investigate the ERK signaling molecules, (b) while at 72 h for the synaptophysin and GAP43 protein measurement by western blot analysis. Band intensities are shown as the bar graph. Moreover, (c) the PC12 morphology was observed after 72 h by using an inverted phase-contrast microscope. (d) The percentage of differentiation and (e) neurite length were calculated. Data represent the means ± SD of three independent experiments. ^#^*p* < 0.05 and ^##^*p* < 0.01 compared with the NGF alone-treated group; ^$$^*p* < 0.01 and ^$$^*p* < 0.01 compared with the combination treatment group. SE: sesamin; NGF: nerve growth factor.

Then, we confirmed this correlation by using JGB1741, a specific SIRT1 inhibitor. The results showed that JGB1741 effectively blocked the action of combination treatment to enhance PC12 differentiation. The levels of phosphorylated ERK1/2 as well as its downstream proteins, GAP43 and synaptophysin, were significantly reduced in JGB1741 treatment compared with combination treatment alone (Figures 8(a) and 8(b)). Meanwhile, the data from morphological observation demonstrated that the effects of combination treatment on cell differentiation and neurite length were also interrupted by the SIRT1 inhibitor (Figures 8(c)–8(e)). Taken together, our results suggest that the potentiation effect of sesamin on NGF-induced neurite outgrowth is involved in SIRT1 protein modulation.

## 4. Discussion

The therapeutic strategy by diminishing the neuronal loss is the main target to alleviate neurodegeneration in neurodegenerative diseases. Meanwhile, neurogenesis is also important for improving neuronal function and cognitive impairment. Recently, the study on phytochemical compounds that can protect neurons or increase neurogenesis holds promise for the treatment of neurodegenerative diseases. Sesamin, a major lignan from sesame seeds and oil, exhibits the neuroprotective effects in various neurodegenerative conditions. Our previous study also demonstrated that sesamin inhibited LPS-induced microglial activation and protected neuronal cell death [9]. Simultaneously, sesamin was found to improve memory and behavior in chronic mild stress-induced memory deficits [18].

Importantly, the brain permeability of sesamin was reported in previous studies. The previous study of Umeda-Sawada demonstrated the sesamin can be detected in rat's brain after oral administration of sesamin at 0.5% (w/w) by high-performance liquid chromatography (HPLC). In the brain, sesamin was first detected at 3 h, peaked at 6 h, and gradually decreased at 9 h [19]. Moreover, Tomimori's study proved the absorption and distribution of orally administered [14C]sesamin at a dose of 5 mg/kg (4.77 MBq/kg) in rats. At 1 h, the radioactivity of [14C]sesamin in the brain and spinal cord was detected [20]. In addition, there are other reports that confirm the blood-brain barrier permeability of sesamin. The neuroprotective effects of sesamin were indicated in many in vivo studies using sesamin oral administration models [21, 22]. The administration of sesamin alleviated blood-brain barrier disruption in mice with traumatic brain injury and reduced the size of brain infarct in ischemic mice [23]. Additionally, the pharmacokinetics of sesamin in humans showed that sesamin was detected in blood at 5 h after ingestion and cleared from the body within 24 h. These data confirmed the safety of sesamin use in humans [24]. From these reports, sesamin has become an interesting phytochemical compound for neurodegenerative disease treatment. However, the effect of sesamin on neurogenesis has not been reported.

Here, we demonstrated the sesamin promoted NGF-induced neurite outgrowth and synaptic connections in PC12 cells. The number of differentiated cells and neurite length were significantly increased in sesamin combined with a low concentration of NGF (20 ng/ml). This result indicated that sesamin enhanced neurogenesis under insufficient NGF condition. Moreover, the combination treatment showed the potentiality to induce neurite outgrowth similar to high NGF (40 ng/ml). This is particularly interesting because the previous study reported that NGF activity was reduced in the basal forebrain cholinergic neurons of AD patients' brains [25]. Thus, the NGF-potentiating activity of sesamin may be a highly useful tool for the induction of neurogenesis in neuropathological conditions. However, sesamin alone does not affect PC12 cell differentiation. This result is consistent with Hamada's study which reported that metabolites of sesamin enhanced the neuronal differentiation, but not parental compounds [2]. Nevertheless, there are several natural compounds that have the effect to potentiate NGF activity, but lack the ability to induce neurite outgrowth by themselves [6].

Next, proteins associated with neurite outgrowth and synaptic formation were further examined. We found that the combination treatment with sesamin and NGF significantly increased GAP43 and SYP expression in both gene and protein levels and the number of synaptic connections. These results were correlated with the percent differentiation and neurite length in our prior experiment. Moreover, other previous studies also demonstrated the effect of GAP43 and SYP in a similar pattern. The previous study indicated that the upregulation of GAP43 augmented NGF-induced neurite outgrowth in PC12 cells. Meanwhile, SYP is characterized as a specific marker for synaptic formation and synaptic connections [2].

For signaling pathway, we indicated that the effect of sesamin on NGF-induced neurite outgrowth was involved in the activation of ERK1/2 pathway. MAPK/ERK1/2 is reported as the direct pathway related to neurite outgrowth. This is confirmed by using U0126, ERK1/2 inhibitor, which significantly decreased GAP43 expression and inhibited the neuronal differentiation and neurite outgrowth [26]. Our results are similar to some compounds that potentiate ERK1/2 activation-dependent NGF stimulation, but cannot induce ERK1/2 pathway on their own [27]. Several references also reported a similar molecular mechanism of sesamin on ERK1/2 modulation [28–30]. For example, Min Zhang's study demonstrated that sesamin itself did not affect the phosphorylation of ERK1/2, but sesamin can modulate the signaling when combined with L-DOPA treatment [29].

Next, we also demonstrated the relationship between the effect of combination treatment on neurite outgrowth and SIRT1 modulation. SIRT1 or NAD+-dependent class III histone deacetylase is an important regulator molecule in neuron cell survival. SIRT1 is an essential regulatory molecule for the neurogenesis process [16]. In the present study, we found that the combination treatment with sesamin significantly increased SIRT1 expression, when compared with NGF treatment alone. This effect of sesamin on SIRT1 modulation was also reported in other studies demonstrating neuroprotective and cardioprotective effects [31, 32]. However, SIRT1 is characterized as a nucleocytoplasmic shuttling protein. Thus, the SIRT1 localization is important to its function. In the brain, SIRT1 is predominantly expressed in the cytoplasm of neural precursor cells and some NeuN + neuron cell types. During the neural precursor cell differentiation, SIRT1 can be transiently shuttled to the nucleus in response to differentiation stimuli and retranslocated to the cytoplasm [33]. However, Sugino's study reported that the cytoplasmic SIRT1 is also essential to promote the NGF-induced neurite outgrowth in PC12 cells [17]. Thus, both cytoplasmic and nuclear SIRT1 are participated in the neurogenesis process. Interestingly, our immunostaining result demonstrated that SIRT1 was upregulated and distributed in both cytoplasm and nucleus of the cells treated with combination treatment, more significantly than those with NGF-treatment alone, while SIRT1 was seen only in the cytoplasm in the control group.

In addition, the correlation of SIRT1 and neurite outgrowth was clearer through using JGB1741, the specific SIRT1 inhibitor, that effectively blocked the combination treatment-induced neurite outgrowth in PC12 cells. These results imply that the potentiating effect of sesamin on NGF function depends on SIRT1 modulation. There are many hypotheses that can explain the effect of SIRT1 modulation on the neurogenesis process. The effect of SIRT1 modulation in the combination treatment might be through the activation of p-ERK1/2, as the relationship between SIRT1 and ERK1/2 was reported in many previous studies [34–36]. In this study, we also demonstrated that inhibition of SIRT1 activity reversed the induction of ERK1/2 phosphorylation by the combination treatment. This result is comparable with other research studies such as Xi Fang's study which reported that downregulation of SIRT1 led to p-ERK1/2 suppression whereas the upregulation of SIRT1 increased p-ERK1/2 activation resulting in improved spatial memory in in vivo model [34]. Another hypothesis is that SIRT1 is involved in the regulation of the cAMP response element-binding protein (CREB) that is the transcription factor essential for neurogenesis and synaptic plasticity [37]. The previous study found that the expression of CREB target is markedly reduced in the brain of SIRT1-KO mice [38].

In summary, sesamin is the compound that renowned for the neuroprotective property in various neuropathological conditions. This study revealed the neurotropic effect of sesamin on the promotion of NGF-induced neurite outgrowth and synaptic connection in PC12 cells under insufficient NGF function via the modulation of SIRT1 protein. Taken together, these data suggest that both neurotrophic and neuroprotective effects of sesamin have broad therapeutic potential in the treatment of neurodegenerative disorders.

## Figures and Tables

**Table 1:** Primers used for gene expression by real-time RT-PCR.

Genes	Sequence (5′-3′): forward (F); reverse (R)	Accession number
SYP	F: AAAGGCCTGTCCGATGTGAAG	NM_012664.3
	R: TCCCTCAGTTCCTTGCATGTG	
GAP43	F: AAGCTACCACTGATAACTCGCC	NM_017195.3
	R: CTTCTTTACCCTCATCCTGTCG	
SIRT1	F: TGGTATTTATGCTCGCCTTGC	NM_001372090.1
	R: CATGAATGCTGAGTTGCTGGA	
ACTB	F: GTAAAGACCTCTATGCCAACA	NM_031144.3
	R: GGACTCATCGTACTCCTGCT	

## Data Availability

The data that support the findings of this study are available from the corresponding author, upon reasonable request.
